# Long-Acting β_2_-Agonists Increase Fluticasone Propionate-Induced Mitogen-Activated Protein Kinase Phosphatase 1 (MKP-1) in Airway Smooth Muscle Cells

**DOI:** 10.1371/journal.pone.0059635

**Published:** 2013-03-22

**Authors:** Melanie Manetsch, Md. Mostafizur Rahman, Brijeshkumar S. Patel, Emma E. Ramsay, Nowshin N. Rumzhum, Hatem Alkhouri, Qi Ge, Alaina J. Ammit

**Affiliations:** 1 Respiratory Research Group, Faculty of Pharmacy, University of Sydney, Sydney, Australia; 2 Woolcock Institute of Medical Research, University of Sydney, Sydney, Australia; National Heart and Lung institute, United Kingdom

## Abstract

Mitogen-activated protein kinase phosphatase 1 (MKP-1) represses MAPK-driven signalling and plays an important anti-inflammatory role in asthma and airway remodelling. Although MKP-1 is corticosteroid-responsive and increased by cAMP-mediated signalling, the upregulation of this critical anti-inflammatory protein by long-acting β_2_-agonists and clinically-used corticosteroids has been incompletely examined to date. To address this, we investigated MKP-1 gene expression and protein upregulation induced by two long-acting β_2_-agonists (salmeterol and formoterol), alone or in combination with the corticosteroid fluticasone propionate (abbreviated as fluticasone) in primary human airway smooth muscle (ASM) cells *in vitro*. β_2_-agonists increased MKP-1 protein in a rapid but transient manner, while fluticasone induced sustained upregulation. Together, long-acting β_2_-agonists increased fluticasone-induced MKP-1 and modulated ASM synthetic function (measured by interleukin 6 (IL-6) and interleukin 8 (IL-8) secretion). As IL-6 expression (like MKP-1) is cAMP/adenylate cyclase-mediated, the long-acting β_2_-agonist formoterol increased IL-6 mRNA expression and secretion. Nevertheless, when added in combination with fluticasone, β_2_-agonists significantly repressed IL-6 secretion induced by tumour necrosis factor α (TNFα). Conversely, as IL-8 is not cAMP-responsive, β_2_-agonists significantly inhibited TNFα-induced IL-8 in combination with fluticasone, where fluticasone alone was without repressive effect. In summary, long-acting β_2_-agonists increase fluticasone-induced MKP-1 in ASM cells and repress synthetic function of this immunomodulatory airway cell type.

## Introduction

Asthma is a chronic airway disease characterized by excessive inflammation. Corticosteroids are widely-used to repress inflammation in asthma and control of asthma symptoms can be improved by combination therapy with long-acting β_2_-agonists [1], [2], [3]. There are a number of mechanisms by which this enhanced anti-inflammatory effect might be achieved at the cellular level: these include control of histone acetylation, increased function of glucocorticoid receptor (GR) or the β_2_-adrenergic receptor, and upregulation of anti-inflammatory proteins (reviewed in [4]). Mitogen-activated protein kinase phosphatase 1 (MKP-1) is one such anti-inflammatory protein whose upregulation is increasingly recognized as an important molecular mechanism responsible for the improved anti-inflammatory action of corticosteroids and long-acting β_2_-agonists in combination.

MKP-1 is an immediate-early gene [5] that is corticosteroid-responsive [6] and contains a cAMP-responsive element (CRE) in its 5′-promoter [7], [8]. We and others have demonstrated that the upregulation of MKP-1 by corticosteroids is responsible, in part, for their anti-inflammatory actions in clinically-relevant airway cell types such as airway smooth muscle (ASM) and bronchial epithelial cells [9], [10], [11], [12], [13], [14]. Moreover, MKP-1 mRNA expression and protein upregulation can be enhanced by long-acting β_2_-agonists [12], [15]. The increased expression of MKP-1 may explain the beneficial effects of β_2_-agonists/corticosteroid combination therapies in the repression of inflammatory gene expression in asthma [12], [16]. However the upregulation of this critical anti-inflammatory protein by long-acting β_2_-agonists and clinically-used corticosteroids has been incompletely examined to date.

To address this gap in knowledge, the aim of this study is to examine MKP-1 mRNA expression and protein upregulation induced by two long-acting β_2_-agonists (salmeterol and formoterol), alone or in combination with the corticosteroid fluticasone propionate (abbreviated as fluticasone). We will assess the anti-inflammatory function of MKP-1 induced by these combination therapies on the synthetic function of primary human ASM cell *in vitro* by measuring repression of interleukin 6 (IL-6) and interleukin 8 (IL-8); two cytokines known to be upregulated in a MAPK-mediated manner [10], [17], [18].

## Materials and Methods

### Cell Culture

Human bronchi were obtained from patients undergoing surgical resection for carcinoma or lung transplant donors in accordance with procedures approved by the Sydney South West Area Health Service and the Human Research Ethics Committee of the University of Sydney. ASM cells were dissected, purified and cultured as previously described by Johnson *et al.* [19]. A minimum of three different ASM primary cell cultures established from individual patients were used for each experiment.

Unless otherwise specified, all chemicals used in this study were purchased from Sigma-Aldrich (St. Louis, MO).

### Western Blotting

MKP-1 was quantified by Western blotting using a rabbit polyclonal antibody against MKP-1 (C-19: Santa Cruz Biotechnology, Santa Cruz, CA), compared to α-tubulin used as the loading control (mouse monoclonal IgG_1_, DM1A: Santa Cruz Biotechnology, Santa Cruz, CA). Primary antibodies were detected with goat anti-mouse or anti-rabbit HRP-conjugated secondary antibodies (Cell Signaling Technology) and visualized by enhanced chemiluminescence (PerkinElmer, Wellesley, MA).

### Real-time RT-PCR

Total RNA was extracted using the RNeasy Mini Kit (Qiagen Australia, Doncaster, VIC, Australia) and reverse transcription performed by using the RevertAid First strand cDNA. MKP-1, IL-6 and IL-8 mRNA levels were measured using real-time RT-PCR on an ABI Prism 7500 (Applied Biosystems, Foster City, CA) with the MKP-1 (DUSP1: Hs00610256_g1), IL-6 (Hs00174131_m1) or IL-8 (Hs00174103_m1) TaqMan® Gene Expression Assays and the eukaryotic 18S rRNA endogenous control probe (Applied Biosystems) subjected to the following cycle parameters: 50°C for 2 min, 1 cycle; 95°C for 10 min, 1 cycle; 95°C for 15 s, 60°C for 1 min, 40 cycles and mRNA expression (fold increase) quantified by delta delta Ct calculations.

### ELISAs

Cell supernatants were collected and stored at −20°C for later analysis by ELISA. IL-6 and IL-8 ELISAs were performed according to the manufacturer’s instructions (BD Biosciences Pharmingen, San Diego, CA).

### MKP-1 siRNA Knock-down

Protein levels of MKP-1 were reduced by RNA interference and the effect on cytokine secretion examined. ASM cells were transiently transfected using nucleofection with 1 µg MKP-1-specific SMART pool siRNA, consisting of a pool of four individual siRNA from Dharmacon (Dharmacon, Lafayette, CO) or a scrambled siRNA control, using methods established in our previous publication [10]. Briefly, ASM cells were transfected with the Nucleofector (Amaxa, Koln, Germany), using the basic kit for primary smooth muscle cells with the manufacturer’s optimized protocol of P-024. ASM cells were plated for 16 h after transfection, before being growth-arrested for a further 24 h. Cells were pretreated for 1 h with formoterol+fluticasone (along with relevant controls) before stimulation for 24 h with TNFα (10 ng/ml). Supernatants were then removed for IL-6 and IL-8 protein secretion by ELISA and lysates utilized for MKP-1 Western blotting (compared to α-tubulin as a loading control).

### Statistical Analysis

Statistical analysis was performed using either Student's unpaired *t* tests, repeated measured ANOVA or two-way ANOVA followed by Bonferroni’s multiple comparison tests. *P* values <0.05 were sufficient to reject the null hypothesis for all analyses.

## Results

### Long-acting β_2_-agonists Increase Fluticasone-induced MKP-1 Protein Upregulation

**Figure 1:**
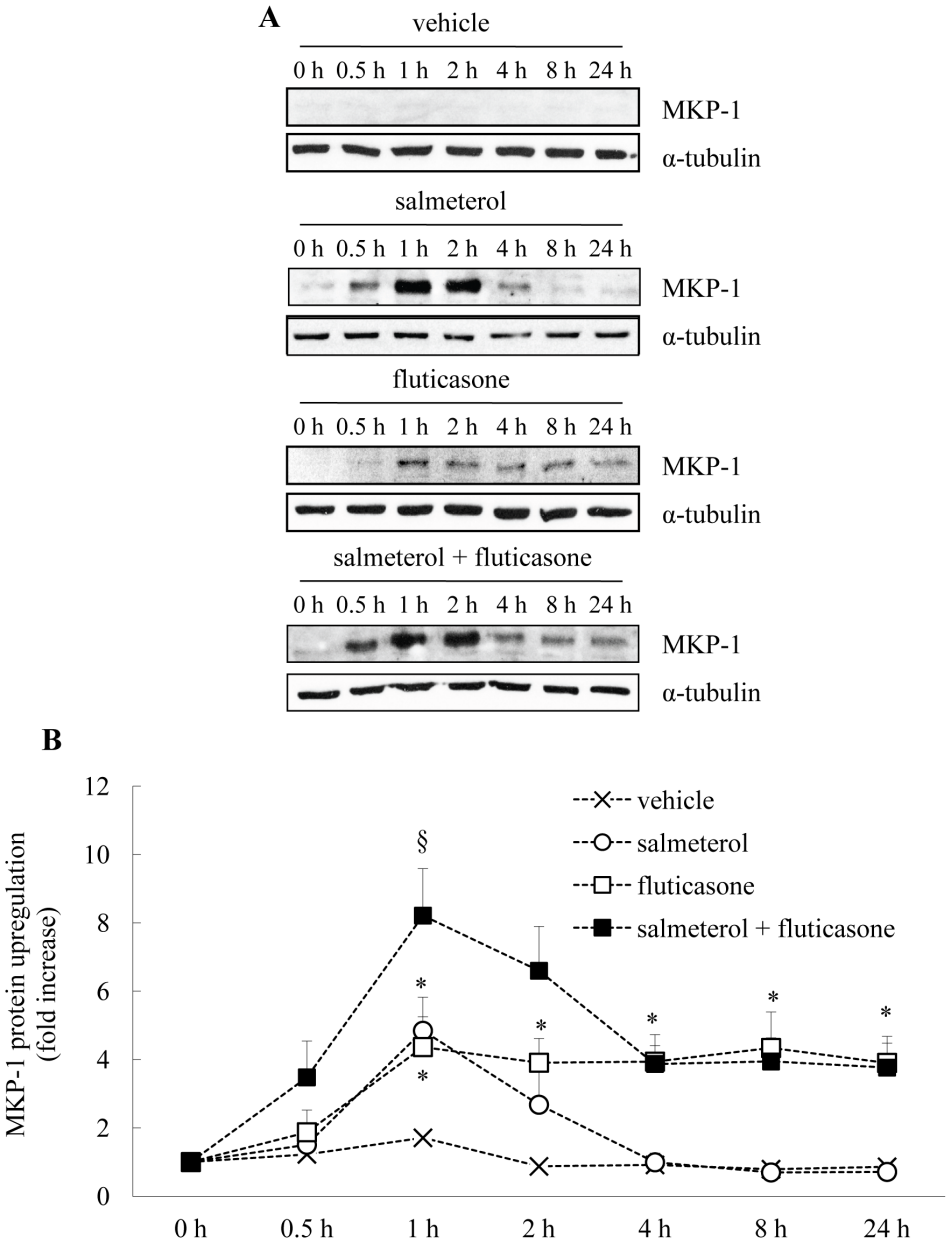
Salmeterol increases fluticasone-induced MKP-1 protein upregulation. (A) Growth-arrested ASM cells were treated with vehicle, salmeterol (100 nM), fluticasone (1 nM), salmeterol (100 nM)+fluticasone (1 nM), for 0, 0.5, 1, 2, 4, 8, and 24 h. MKP-1 protein (molecular weight:∼39 kDa) was quantified by Western blotting, using α-tubulin as the loading control (molecular weight: 55∼kDa), where (A) illustrates representative Western blots and (B) demonstrates densitometric analysis where results are expressed as fold increase over 0 h (mean+SEM values from n = 6–8 primary ASM cell cultures). Statistical analysis was performed using two-way ANOVA then Bonferroni's post-test (where * indicates a significant effect of salmeterol or fluticasone on MKP-1 protein upregulation, compared to vehicle-treated cells, and § indicates a significant effect of salmeterol on fluticasone-induced MKP-1 (*P*<0.05)).

**Figure 2:**
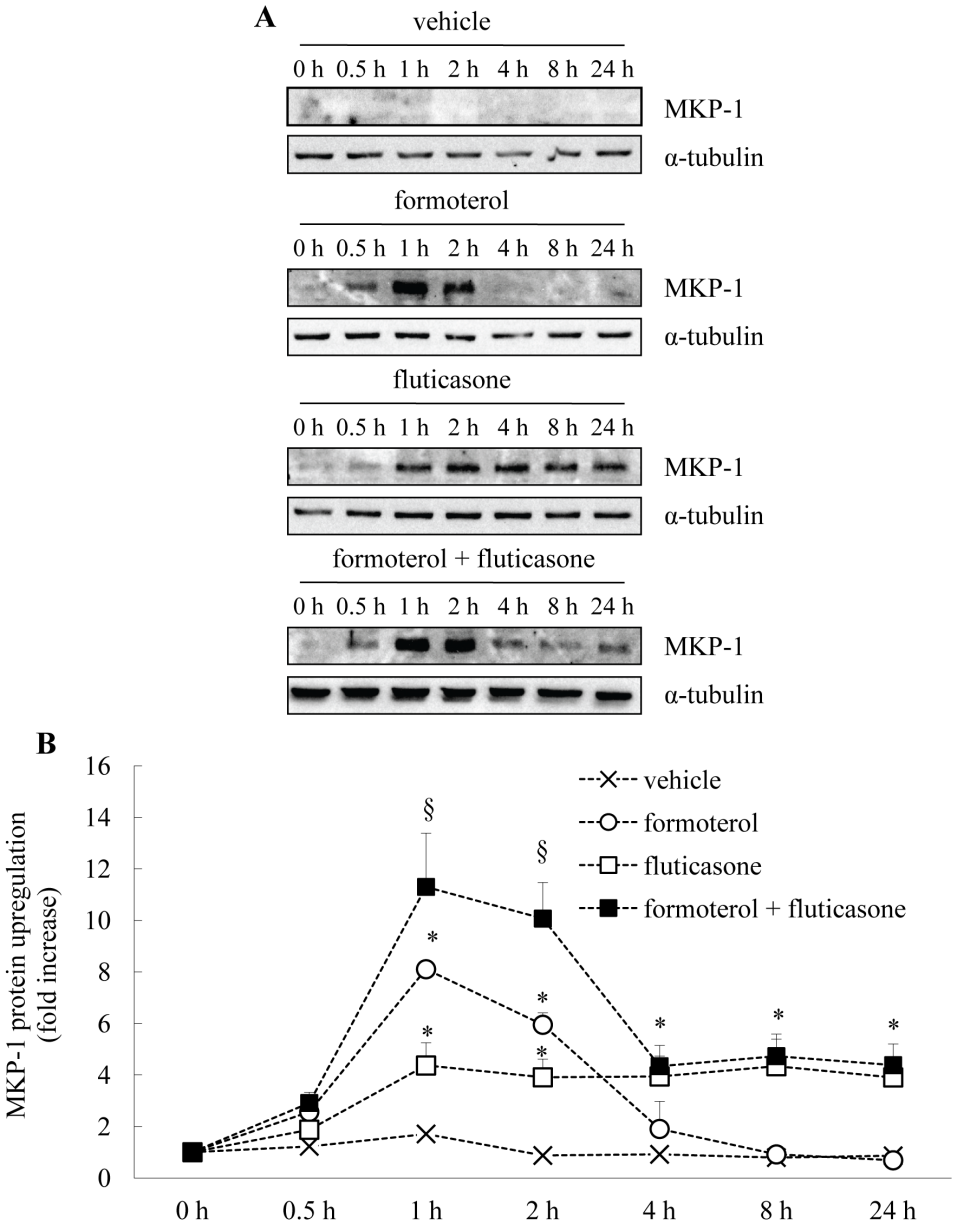
Formoterol increases fluticasone-induced MKP-1 protein upregulation. (A) Growth-arrested ASM cells were treated with vehicle, formoterol (10 nM), fluticasone (1 nM), formoterol (10 nM)+fluticasone (1 nM), for 0, 0.5, 1, 2, 4, 8, and 24 h. MKP-1 protein (molecular weight:∼39 kDa) was quantified by Western blotting, using α-tubulin as the loading control (molecular weight:∼55 kDa), where (A) illustrates representative Western blots and (B) demonstrates densitometric analysis where results are expressed as fold increase over 0 h and results for fluticasone (from Figure 1B) shown graphically for comparative purposes (mean+SEM values from n = 3–8 primary ASM cell cultures). Statistical analysis was performed using two-way ANOVA then Bonferroni's post-test (where * indicates a significant effect of formoterol or fluticasone on MKP-1 protein upregulation, compared to vehicle-treated cells, and § indicates a significant effect of formoterol on fluticasone-induced MKP-1 (*P*<0.05)).

Although MKP-1 is known to be corticosteroid-responsive [10], [20] and increased by cAMP-mediated signalling [20] in ASM cells, the upregulation of this critical anti-inflammatory protein by long-acting β_2_-agonists and clinically-used corticosteroids has been incompletely examined to date. To address this, we examine here for the first time, the effect of treating ASM cells with fluticasone, alone or in combination with the long-acting β_2_-agonists - salmeterol (100 nM: Figure 1) or formoterol (10 nM: Figure 2). These concentrations of β_2_-agonists were chosen for the current study as they had been used previously in *in vitro* investigations of MKP-1 gene expression by Kaur *et al.* [12].

We first examined the temporal kinetics of MKP-1 protein upregulation in ASM cells treated for up to 24 h with salmeterol or fluticasone, alone or in combination (compared to vehicle-treated controls). As shown in Figure 1A, salmeterol alone induced rapid, but transient upregulation of MKP-1 with a peak at 1 h. In contrast, fluticasone alone induced a sustained increase in MKP-1 protein upregulation; in accordance with our earlier report [10]. This was confirmed by densitometry (as shown in Figure 1B) where salmeterol induced significantly greater levels of MKP-1 protein (4.8±1.0-fold) than cells treated with vehicle alone (1.7±0.2-fold) at 1 h (*P*<0.05). While MKP-1 upregulation induced by salmeterol was transient (returning back to basal levels at 4 h), fluticasone significantly increased MKP-1 protein levels by 4.4±0.9-fold at 1 h and this increase was sustained for up to 24 h (3.9±0.8-fold)((*P*<0.05). When we then treat cells with salmeterol in combination with fluticasone (Figure 1A), the temporal pattern of MKP-1 protein upregulation reflects the combined contribution of the β_2_-agonist and corticosteroid. This is best demonstrated by densitometric analysis (Figure 1B), where both drugs in combination are shown to act in an additive manner and salmeterol added in combination with fluticasone induces significantly greater levels of MKP-1 protein (8.2±1.4-fold), than fluticasone alone at 1 h (4.4±0.9-fold) (*P*<0.05).

We then compared the effect of formoterol on fluticasone-induced MKP-1 (Figure 2). In corroboration of our earlier report [20], formoterol alone induces a transient increase in MKP-1 upregulation with a peak at 1–2 h (Figure 2A). Importantly, Western blotting (Figure 2A) revealed that when formoterol and fluticasone are added in combination, the peak of MKP-1 upregulation that appears at 1–2 h after the long-acting β_2_-agonist and enhances the sustained pattern of MKP-1 upregulation observed with fluticasone treatment alone. This is supported by densitometric analysis where formoterol significantly increased fluticasone-induced MKP-1 at 1 h (11.3±2.1-fold) and 2 h (10.1±1.4-fold), compared to fluticasone alone at the same time points (4.4±0.9- and 3.9±0.7-fold, respectively) (*P*<0.05).

### Long-acting β2-agonists Increase Fluticasone-induced MKP-1 mRNA Expression

**Figure 3:**
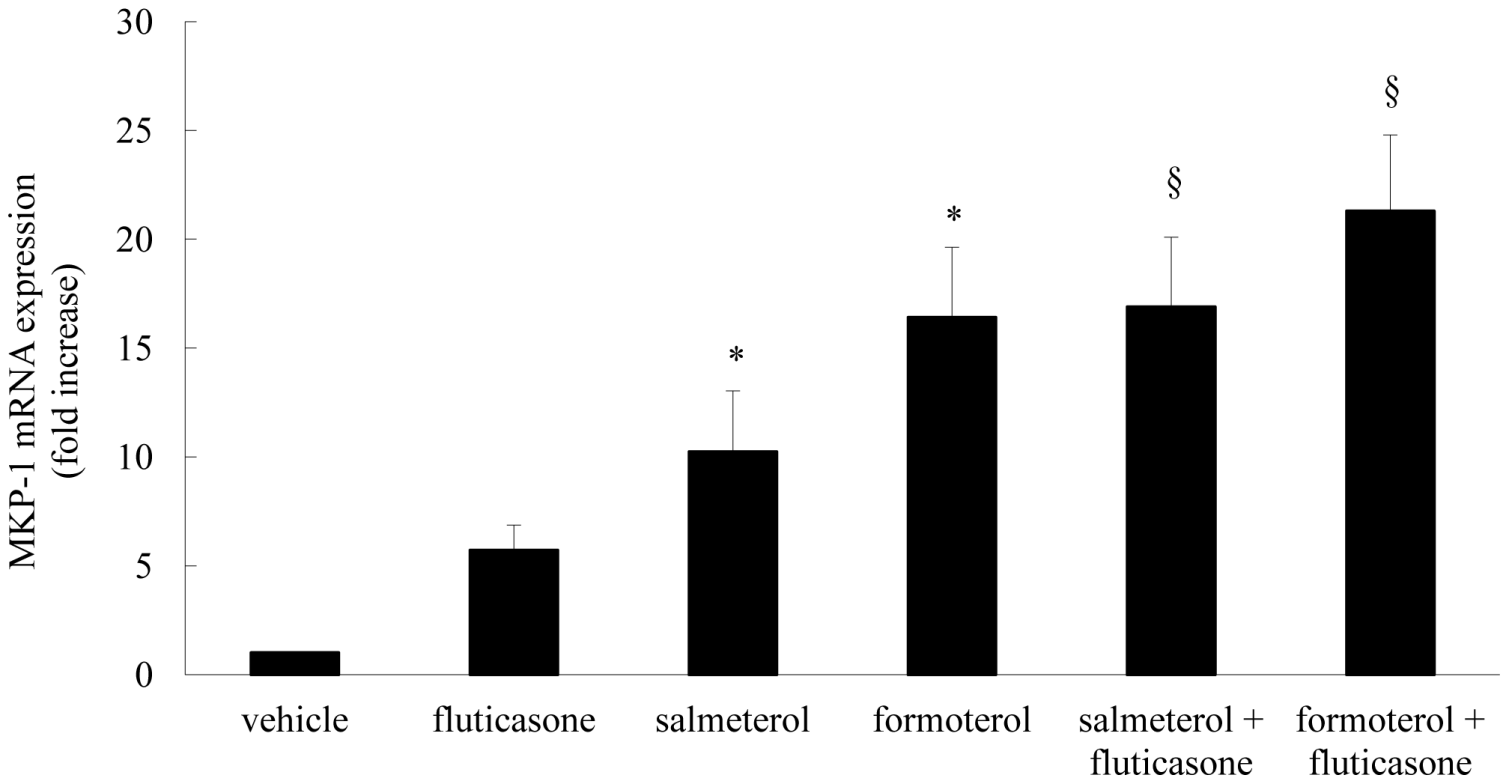
Long-acting β_2_-agonists increase fluticasone-induced MKP-1 mRNA expression. Growth-arrested ASM cells were treated for 1 h with vehicle, fluticasone (1 nM), salmeterol (100 nM), formoterol (10 nM), salmeterol (100 nM)+fluticasone (1 nM), formoterol (10 nM)+fluticasone (1 nM). MKP-1 mRNA expression was quantified by real-time RT-PCR and results expressed as fold increase compared to vehicle-treated cells (mean+SEM values from n = 9 primary ASM cell cultures). Statistical analysis was performed using repeated measures ANOVA with Bonferroni’s multiple comparison test (where * denotes a significant increase in MKP-1 mRNA expression and § denotes a significant effect of the β_2_-agonists on fluticasone-induced MKP-1 mRNA expression (*P*<0.05)).

MKP-1 is an immediate-early gene and we previously reported that the corticosteroid dexamethasone and the long-acting β_2_-agonist formoterol rapidly increased MKP-1 gene expression by 1 h [20]. In the current study we extend these observations and perform a side-by-side comparison of MKP-1 mRNA upregulation in response to the long-acting β_2_-agonists salmeterol and formoterol, and the corticosteroid fluticasone, alone and in combination. While MKP-1 gene expression induced by fluticasone (5.7±1.2-fold, compared to vehicle-treated cells) trended towards significance, salmeterol and formoterol added alone significantly increased MKP-1 gene expression by 10.2±2.8-fold and 16.4±3.2-fold, respectively (*P*<0.05). Importantly, when long-acting β_2_-agonists were added in combination with fluticasone they significantly augmented steroid-induced MKP-1 mRNA expression (*P*<0.05). As shown in Figure 3, salmeterol increased MKP-1 expression to 16.4±3.2-fold and formoterol to 21.3±3.5-fold, compared to fluticasone alone (5.7±1.2-fold, respectively) (*P*<0.05).

### Effect of Long-acting β_2_-agonists on IL-6 and IL-8 mRNA Expression and Secretion

**Figure 4:**
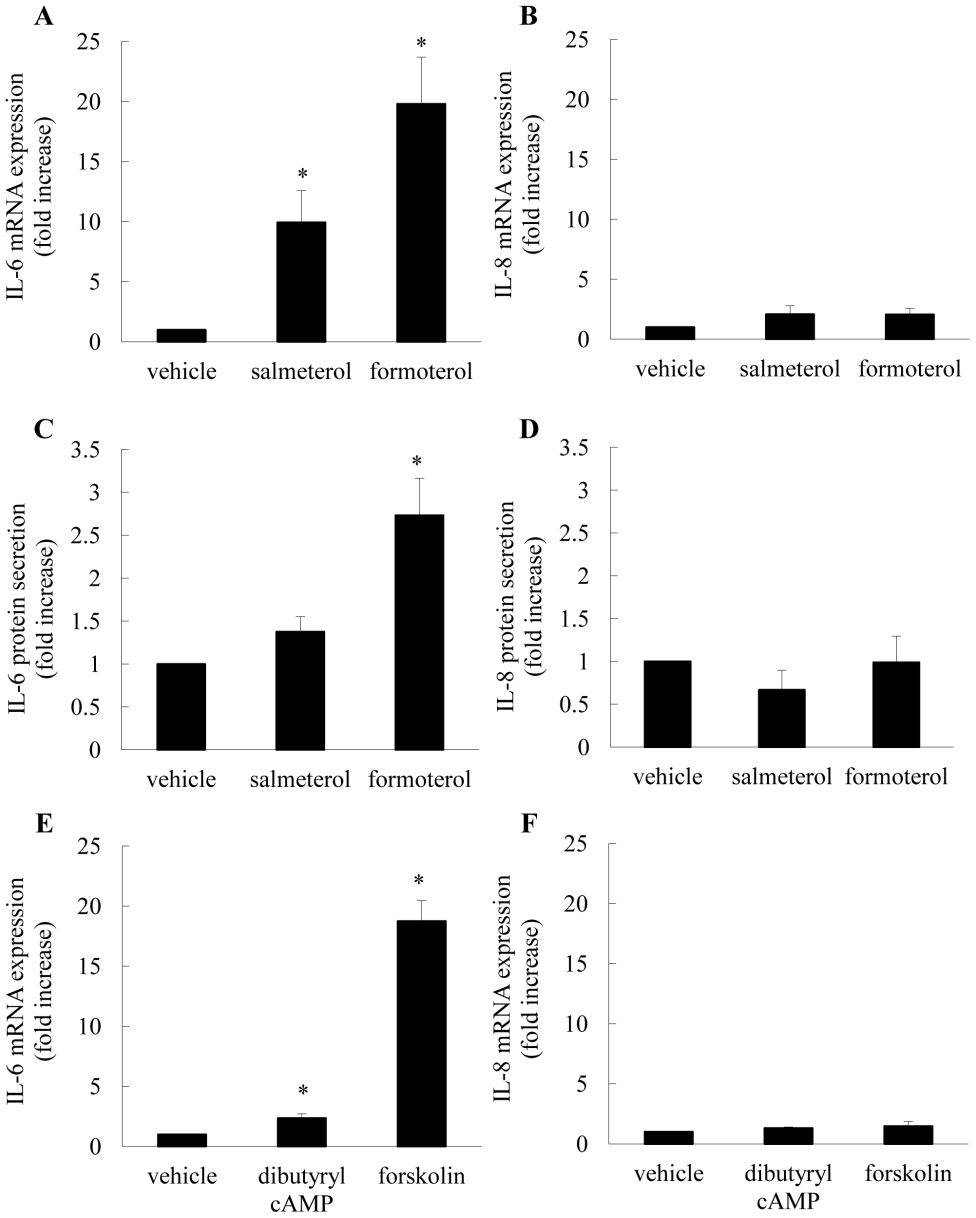
Effect of long-acting β_2_-agonists on IL-6 and IL-8 mRNA expression and secretion. (A, B, C, D) Growth-arrested ASM cells were treated with vehicle, salmeterol (100 nM) or formoterol (10 nM). (A) IL-6 and (B) IL-8 mRNA expression was quantified by real-time RT-PCR at 1 h and results expressed as fold increase compared to vehicle-treated cells (mean+SEM values from n = 9 primary ASM cell cultures). (C) IL-6 and (D) IL-8 secretion was measured by ELISA at 24 h and results are expressed as fold increase compared to vehicle-treated cells (where IL-6 and IL-8 protein levels in cells treated with vehicle were 295.0±34.8 and 26.3±4.6 pg/ml, respectively (mean+SEM values from n = 6 primary ASM cell cultures)). Statistical analysis was performed using repeated measures ANOVA with Bonferroni’s multiple comparison test (where * denotes a significant increase in mRNA expression or secretion (*P*<0.05)). (E, F) Growth-arrested ASM cells were treated with vehicle, dibutyryl cAMP (1 mM), forskolin (10 µM) and (E) IL-6 and (F) IL-8 mRNA expression was quantified by real-time RT-PCR at 1 h. Results are expressed as fold increase compared to vehicle-treated cells (mean+SEM values from n = 3 primary ASM cell cultures). Statistical analysis was performed using the Student's unpaired *t* test where * denotes a significant increase in mRNA expression or secretion (*P*<0.05).

As the final aim of the current study was to demonstrate the anti-inflammatory function of MKP-1 on ASM synthetic function by measuring repression of IL-6 and IL-8 secretion induced by TNFα (see next section), it was first important to examine the effects of β_2_-agonists alone on secretion of these cytokines by ASM cells. Interestingly, IL-6 (like MKP-1) is cAMP-responsive. In this way, we show that salmeterol and formoterol significantly enhance IL-6 mRNA expression at 1 h (Figure 4A) and that formoterol increases IL-6 secretion from ASM cells at 24 h (Figure 4C) (*P*<0.05). These protein secretion results are in confirmation of our earlier study [21] and in accord with the relative ability of these β_2_-agonists to elevate cAMP in ASM cells [21]. The involvement of cAMP/adenylate cyclase-mediated pathway in IL-6 mRNA expression is further substantiated by treatment of ASM cells with the cell-permeable cAMP analogue, dibutyryl cAMP, and the adenylate cyclase activator, forskolin. As shown in Figure 4E, dibutyryl cAMP and forskolin increased IL-6 mRNA expression by 2.4±0.4-fold and 18.8±1.7-fold, respectively, compared to vehicle control (*P*<0.05). IL-8, in contrast, is not regulated by cAMP and thus long-acting β_2_-agonists do not increase IL-8 mRNA expression (Figure 4B), nor protein secretion (Figure 4D) in ASM cells. Moreover, dibutyryl cAMP and forskolin are without effect on IL-8 mRNA expression (Figure 4F).

### The Effect of Long-acting β_2_-agonists and Fluticasone, Alone or in Combination, on Repression of TNFα-induced IL-6 and IL-8 Secretion

**Figure 5:**
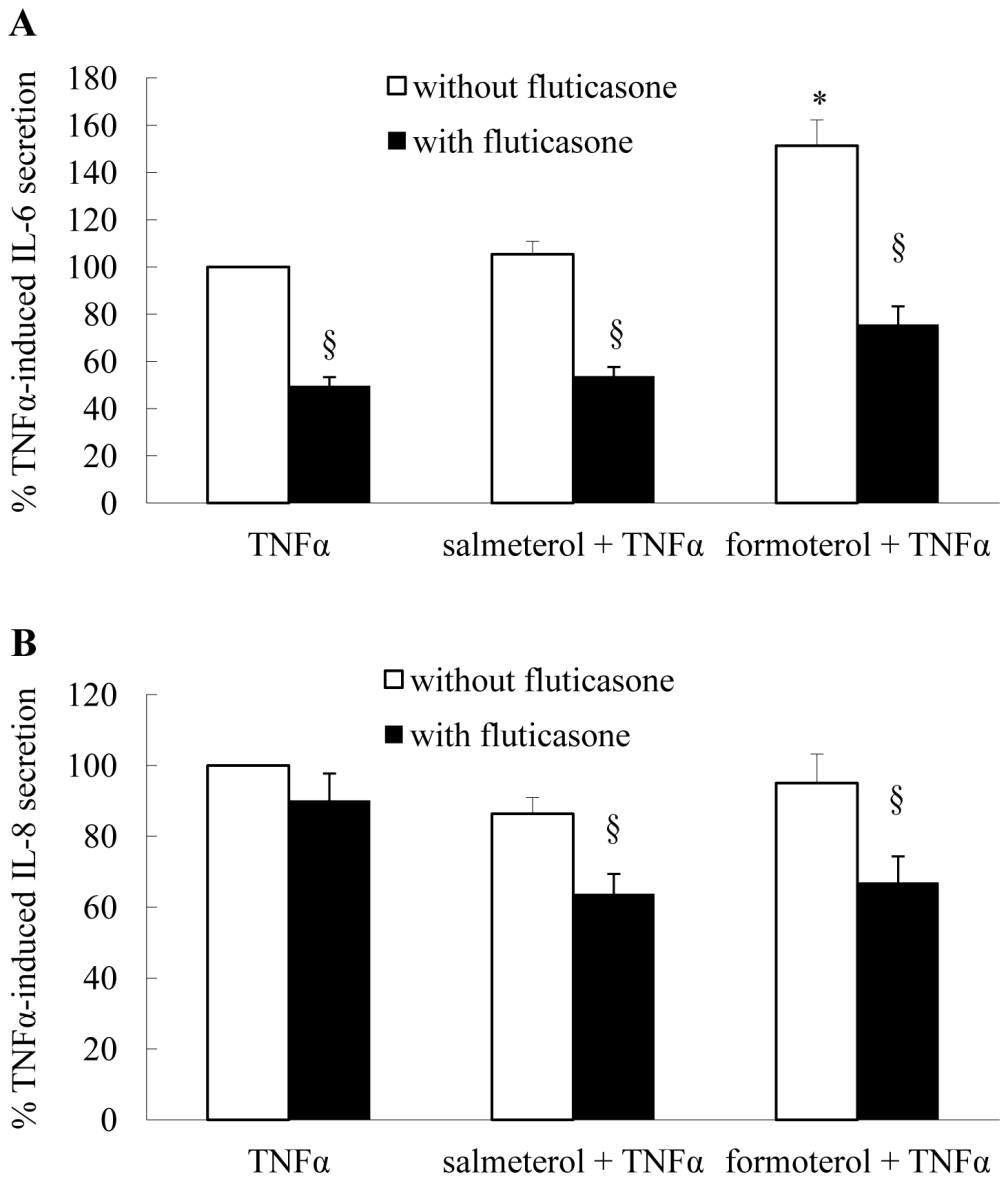
The effect of long-acting β_2_-agonists and fluticasone, alone or in combination, on repression of TNFα-induced IL-6 and IL-8 secretion. Growth-arrested ASM cells were pretreated for 1 h with vehicle, salmeterol (100 nM) or formoterol (10 nM), alone or in combination with fluticasone (1 nM), before stimulation for 24 h with TNFα (10 ng/ml). (A) IL-6 and (B) IL-8 secretion was measured by ELISA and results expressed as (A) % TNFα-induced IL-6 secretion or (B) % TNFα-induced IL-8 secretion (where TNFα-induced IL-6 and IL-8 protein secretion was 5153.9±618.9 and 5913.1±480.0 pg/ml, respectively (mean+SEM values from n = 6 primary ASM cell cultures)). Statistical analysis was performed using repeated measures ANOVA with Bonferroni’s multiple comparison test where * denotes a significant increase in IL-6 secretion and § denotes a significant repression of TNFα-induced cytokine secretion (*P*<0.05).

Finally, the effect of MKP-1 upregulation induced by β_2_-agonists, alone or in combination with the corticosteroid fluticasone, was demonstrated by pretreating ASM cells for 1 h before stimulation with TNFα. As shown in Figure 5A, and explained by the cAMP-responsiveness of IL-6 (described earlier), formoterol significantly enhanced TNFα-induced IL-6 secretion. Despite this, when β_2_-agonists were added in combination with fluticasone they still significantly repressed TNFα-induced IL-6 (*P*<0.05). IL-8, on the other hand, is not cAMP-responsive; thus, β_2_-agonists did not significantly increase TNFα-induced IL-8 (Figure 5B). Interestingly, fluticasone alone did not repress TNFα-induced IL-8, while salmeterol, or formoterol, in combination with fluticasone significantly inhibited IL-6 secretion (Figure 5B). The combination of the long-acting β_2_-agonists with fluticasone, has a statistically greater effect than fluticasone alone on TNFα-induced IL-8 secretion (*P*<0.05). Taken together, our results indicate that long-acting β_2_-agonists increase fluticasone-induced MKP-1 in an additive manner and in this way MKP-1 acts to repress cytokine secretion from ASM cells.

**Figure 6:**
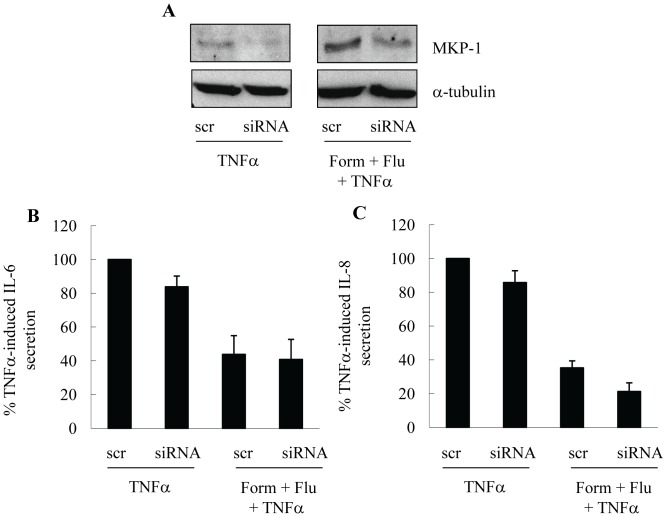
Effect of MKP-1 siRNA. ASM cells were transiently transfected using nucleofection with scrambled control or MKP-1 siRNA, growth-arrested, then pretreated for 1 h with formoterol (10 nM)+fluticasone (1 nM) (along with relevant controls) before stimulation for 24 h with TNFα (10 ng/ml). Supernatants were then removed for IL-6 and IL-8 protein secretion by ELISA and lysates utilized for MKP-1 Western blotting (compared to α-tubulin as a loading control). (A) illustrates a representative Western blot, while (B, C) demonstrates results expressed as % TNFα-induced (B) IL-6 or (C) IL-8 secretion in cells transfected with scrambled control siRNA (mean+SEM values from n = 6 primary ASM cell cultures).

In preliminary studies, we then knocked-down MKP-1 with siRNA in an attempt to reverse the repression of cytokine secretion by formoterol and fluticasone, in combination. Although MKP-1 siRNA successfully reduced protein levels of MKP-1 in ASM cells (Figure 6A) we were unable to reverse the inhibition of TNFα-induced IL-6 (Figure 6B) or IL-8 (Figure 6C) secretion by formoterol and fluticasone. Investigations are now ongoing to examine the hypothesis that in the absence of MKP-1, the p38 MAPK-dependent RNA destabilizing factor – tristetraprolin (TTP) – may serve in a compensatory manner to repress inflammation. However further studies will be required to fully understand how these critical anti-inflammatory molecules are intrinsically intertwined and act together as a fail-safe mechanism to regulate inflammation in health and disease.

## Discussion

This study demonstrates that long-acting β_2_-agonists increase fluticasone-induced MKP-1 mRNA expression and protein upregulation in ASM cells and together these asthma drugs have significant effects on cytokine secretion from ASM, an important immunomodulatory cell type in asthma.

Long-acting β_2_-agonists mediate their actions via interaction with the β_2_-adrenergic receptor, activation of adenylate cyclase and production of cAMP. Gene transcription is then induced in genes that contain cAMP-responsive elements (CRE) in their 5′-promoter. MKP-1 has a CRE-containing promoter [7], [8] and we recently reported that the long-acting β_2_-agonist formoterol induced MKP-1 mRNA and protein expression in a cAMP-mediated manner via the β_2_-adrenergic receptor-protein kinase A pathway [15]. MKP-1 gene expression and protein upregulation was rapid and peaked by 1 h [15]. We confirm these studies here and extend them to show that another commonly-used long-acting β_2_-agonist, salmeterol, also rapidly upregulates MKP-1 in a similar manner. Taken together with the demonstration that MKP-1 can be upregulated by cAMP analogues [20] these results present a unifying explanation that agonists that act to increase cAMP will increase MKP-1. Indeed, this has proven to be the case for sphingosine 1-phosphate (S1P), a bioactive sphingolipid known to be elevated in asthma [22]. S1P acts via G_s_-coupled receptors to increase cAMP [22] and we recently demonstrated that S1P increased MKP-1 in a cAMP-dependent manner to serve an important role a negative feedback controller and restrain the extent and duration of pro-inflammatory cellular signalling [20].

Importantly, when long-acting β_2_-agonists are added in combination with the corticosteroid fluticasone they significantly and rapidly upregulated MKP-1 protein and mRNA expression. The temporal pattern of MKP-1 protein upregulation reflects the additive contribution of each asthma drug. That is, β_2_-agonists rapidly, but transiently, upregulates MKP-1 at 1–2 h, while corticosteroids induced a sustained increase in MKP-1 protein. Thus, when added together they upregulate MKP-1 early (by 1 h) but in a sustained manner. This additive upregulation was supported by mRNA levels. These results suggest a molecular basis for the added anti-inflammatory effects of both asthma drugs in combination.

To support the above assertion that increased expression of MKP-1 may explain the beneficial effects of β_2_-agonists/corticosteroid combination therapies in the repression of inflammatory gene expression in asthma [16], we performed *in vitro* studies measuring secretion of IL-6 and IL-8 from ASM cells after stimulation with TNFα. It is important to note that IL-6 is a cAMP-responsive gene. The 5′-promoter for IL-6 contains a CRE [21] and can be induced by cAMP analogues, as well as short-acting and long-acting β_2_-agonists [21], [23]. Herein we confirmed the cAMP-responsiveness of IL-6 by demonstrating significant IL-6 mRNA expression induced by the cell-permeable analogue, dibutyryl cAMP, as well as the adenylate cyclase activator, forskolin. In contrast, cAMP is only a weak activator of IL-8 in ASM cells [24] and, as highlighted by Holden *et al*. in lung epithelial cells [25], the promoter for IL-8 does not contain CRE consensus sequences [26] and that transcriptional regulation may involve cross-talk between transcription factors. Our current study demonstrates the lack of effect of cAMP-elevating agents on IL-8 expression in ASM cells.

Thus, due to the cAMP-dependence of IL-6 expression, the long-acting β_2_-agonist formoterol significantly enhanced TNFα-induced IL-6 secretion. This corroborated our earlier report [21] and underscored the importance of the study by Holden *et al.* [25] where the authors stated that enhancement of IL-6 (and IL-8) release by β_2_-agonists may contribute to the deleterious effects of β_2_-agonist used monotherapy. Notably, when added in combination with fluticasone, β_2_-agonists significantly repressed IL-6 secretion, but not to a greater extent than fluticasone alone. These results are in agreement with Holden *et al.* where increased inflammatory cytokine released was reversed by corticosteroid action [25].

The repressive effect on TNFα-induced IL-8 were more clear-cut, as long-acting β_2_-agonists added on their own did not increase IL-8 expression. Importantly, we observed that fluticasone alone was without repressive effect on TNFα-induced IL-8 secretion, but that β_2_-agonists added in combination significantly repressed TNFα-induced IL-8. This enhanced repressive effect of β_2_-agonists and corticosteroids correlates with the increased MKP-1 expression by both asthma drugs in combination. Why the combined effects of LABA/fluticasone on MKP-1 result in an additive suppressive effect on IL-8, but not on IL-6, cannot be completely delineated at this stage, but may reflect the relative MAPK-dependent contribution to IL-6 and IL-8 secretion. However, our *in vitro* results support those recently published *in vivo* in mild asthmatics [27], where formoterol in combination with corticosteroids improved clinical symptoms (FEV_1_) and reduced IL-8 in a manner linked to MKP-1 upregulation. Interestingly, it has been previously shown by Knobloch *et al.* [28] that the corticosteroid dexamethasone suppresses IL-8 in ASM cells of non-smokers and smokers, but not in those of COPD; whether MKP-1 is involved warrants further investigation.

Our preliminary studies have raised the hypothesis that in the absence of MKP-1 (knocked-down by siRNA), the p38 MAPK-dependent TTP may be upregulated and serve as a compensatory anti-inflammatory factor. TTP is an immediate-early gene [29] that is rapidly and robustly expressed via p38 MAPK signalling [30], [31], [32], [33]. Moreover, TTP’s function as an mRNA destabilising factor is dictated by phosphorylation on two key serine residues (S52 and S178). Further studies investigating the regulation of the RNA destabilising factor TTP by MKP-1, and *visa versa*, in ASM cells are warranted.

In summary, long-acting β_2_-agonists increase fluticasone-induced MKP-1 in ASM cells and together these asthma drugs have significant repressive effects on cytokine secretion from ASM cells. This study further substantiates the anti-inflammatory role played by MKP-1 as an important molecular mechanism responsible for the improved anti-inflammatory action of corticosteroids and long-acting β_2_-agonists in combination in asthma.
